# Observations on the Lichen Islandicus

**Published:** 1808-01

**Authors:** 


### *To the Editors of the Medical and Physical Journal.*

GENTLEMEN,

AS the public attention has lately been much attracted towards the Lichen Islandicus, and its anti-phthisical powers been much extolled, I flatter myself that the following judicious observations, by Professor PROUST, on this subject, may not prove unacceptable to your readers.

After having witnessed a season of scarcity, observes this learned Naturalist, in which many of the poor perished through mere want, it becomes the duty of every humane man to enter upon a new and accurate research into the vegetable kingdom; in order, if possible, to discover some substance, which may serve as aliment, and which may have hitherto escaped our observation.

The Lichens, for example, of which innumerable species cover the rocks throughout Spain, and which supply the inhabitants of Lapland and Iceland with the principal part of their sustenance, might, perhaps, be advantageously applied to this purpose.

Don Mariano la Gasca, celebrated for his discoveries in botanical science, has lately turned his attention to this question. He discovered, in the neighbourhood of the monastery of Harvas, situated at a considerable height in the mountains, which divide the province of Leon from that of Asturias, the same species of Lichen as that from which the Icelanders prepare a kind of bread, equally consistent as that made from wheaten flower. He was also informed, that it grew in great abundance in several different parts of the latter of these provinces.

It is well known, that this moss is indigenous in many of the countries of Europe; it is mentioned in every botanical treatise, and several naturalists have made this tribe of vegetables the particular object of their investigation. It is, nevertheless, truly astonishing, that though they could not be ignorant of the purposes to which it is applied in Iceland, they have, nevertheless, considered it only in a medicinal point of view.

Yet, after all that has been written on this subject, its pectoral qualities are extremely doubtful; while, on the contrary, as affording a wholesome and nutritious aliment, its advantages are incontestible. How, in fact, can we view with indifference a production, which furnishes to the inhabitants of the cantons where it grows, a healthy and agreeable agreeable food, which requires no culture; of which the preparation is as easy as that of potatoes, and which is not, in any respect, inferior to this vegetable.

If we consider, on the contrary, its medicinal qualities, we may inquire to what are they reducible; or even those belonging to all the plants that have been dignified with the name of pectoral? It must be acknowledged, if we are candid, that they owe their boasted virtues to mere illusion; at least, we may as well rank among medicines succulent viands, bread, potatoes, sugar, gums, fruits, and all esculent vegetables, because they possess mucilaginous, pectoral, and demulcent qualities. We may farther inquire, if the pectoral virtues of the lichen rest on any other foundation than those imaginary qualities, with which the doctrine of sympathies has infected therapeutics for more than three centuries?

Is it not, moreover, from the extravagant idea of marks, or resemblances, that certain plants are considered as specifics against particular diseases; as, for example, the agaric, the misletoe of the oak, the peony, &c. collected during the wane of the moon, against affections of the brain; lung-wort, for diseases of the chest; spleen-wort, for those of the spleen; and so many others, the miraculous virtues of which are recorded in the works of Porta, Crellius, &c.?

Naturalists, of the fifteenth century, imagined, from the vague resemblances sometimes discoverable between certain parts of the human body, and the plants we have mentioned, that Providence designed to point them out to man as specifics, in diseases of those organs; and as several species of lichens, dandelion, &c. are marked, so as to bear some kind of resemblance to diseased lungs, they were naturally led to regard them as a beneficent analogy; and that they had only to avail themselves of such indications, in order to arrest the progress of the diseases of this organ.

While modern physicians reject such antiquated fables, do they display greater wisdom, with respect to the species of lichen now under consideration, to which they ascribe innumerable virtues equally ill-founded? If we examine the various medical publications which treat of this moss, which is affirmed to possess aperitive, desiccative, and pectoral qualities, we should be induced to suppose it an universal panacea, against all the maladies to which human nature is incident. It is advantageously employed, they tell us, in phthisis, asthma, inveterate coughs, bilious vomitings, diarrhœa, and dysentery, as well as in gonorrhœa, ulcers of the uterus, hæmorrhoidal affections, &c. &c. In fact, were it possessed of a fourth part of these virtues, observes Amoreux of Montpelier, such a specific might justly be reckoned a gift from the gods.

Willemet, of Nancy, speaks of it in similar terms. My own observation and experience, says he, warrant me to affirm, that it possesses none of those virtues for which it has been so extravagantly extolled in diseases of the pulmonary organs. But let us return to the consideration of those qualities, which render it more worthy of our attention.

Don Mariano la Gasca having sent me a specimen of the lichen, I immediately entered on the examination of its nutritive qualities, without taking into consideration its properties as a dyeing material; since it is already well known that the shades which it imparts to wool, are very indifferent.

To enable us, however, to form a judgment respecting this plant, it appears to me proper succinctly to detail what travellers have related, respecting the uses to which it is applied in Iceland and Lapland.

These details, we observe, the learned Professor has chiefly drawn from the apparatus medicaminum of Murray, which contains every thing yet known in Europe concerning it.

The Icelanders, according to Troilus, make excursions of one or two weeks into the districts where the lichen abounds, and bring it home in bags; in which it is kept till about to be used, when they wash and reduce it to flour. Two tons of this powder, if we may rely on the opinion of Olafer, are equally nourishing as one ton of wheaten flour. After soaking it a whole day in water, to deprive it of its bitterness, they boil it in whey, till it is reduced to a jelly; and eat it, either warm or cold, with milk, or a fresh portion of whey.

The Laplanders boil the lichen in one or two waters, which they throw away; after which they wash it in cold water, and then bruise and boil it in milk. This mess is seasoned with salt. Analysis, however, shows us, that by this process a considerable portion of the nourishing substance is lost.

Some Swedish botanists travelling in Lapland, during the summer of 1788, when the north of Germany and the western parts of Bothnia laboured under a dreadful scarcity, supported themselves on the moss during fourteen days.

days. They macerated it for a single night in warm water, and in the morning boiled it with milk.

In Carniola, they reckon that animals fed on this lichen, are sooner fattened than by any other food. With this view, they drive lean cattle and horses to places wherein it abounds; and in less than four weeks, find them plump and fat.

According to the celebrated Pallas, the pastoral tribes of southern Nessia support themselves on the lichen, when all other aliments fail them.

The above authorities, which, if necessary, might have been greatly multiplied, are sufficient to show the utility of the lichen as an article of food; but they throw no light on its nature or constituent principles; neither are we informed, whether it is before or after being reduced to flour that it is soaked, in order to deprive it of its bitterness; which is essential to be known by those who would apply it to any useful purpose. The following analysis will, however, professor Proust flatters himself, supply this omission.

After separating the moss and the fragments of wood, the next step is to rub it strongly, for a few seconds, in water, in order to free it from any portion of earth, or other impurities, which are frequently found adhering to its stems.

When immersed in cold water, for a few seconds, the lichen resumes its original colour and freshness. One pound of dried moss, treated in this way, and afterwards wiped with a cloth, weighs two pounds two ounces; from which we may conclude, that the green lichen loses nearly one-half of its weight by desiccation.

This moss, kept under water for three or four days, imparts to it a pale fawn colour, but gives out none of its bitter principle; to obtain this, the plant must be previously bruised. In fact, it is not usual for plants, in an entire state, to yield their juice to water; otherwise they would be exposed, while growing, to be so much injured by rain and dews, as to prove destructive to their health and vigour. Nature, however, takes care to prevent such accidents. We may then judge that those travellers, who assert the Icelanders deprive the lichen of its bitterness, merely by soaking it in water, have not sufficiently attended to the practice of these people, who first cut it into small pieces; being fully assured, from experience, that they could not otherwise succeed in depriving it of this principle.

 When

When the lichen is pulverised, and thrown into cold water, a bitter juice, of a light yellow colour, separates from it in less than three hours; its bitterness very much resembles that of succory. Though this bitter principle be destitute of any aromatic quality, it is not particularly nauseous; and the plant is, in a great measure, deprived of it, when subjected to a slight boiling. When it has been infused in cold water for more than twelve hours, it loses about three centiemes of its weight. If hot water be employed, it loses from five to six per cent. and is, at the same time, deprived of a portion of its nutritive principle; but this must appear of little consequence, when we consider that water, when heated from twenty to twenty-five degrees, deprives it of its bitter principle much more promptly than when used cold.

This bitterness resides in what is termed by chemists the *extractive principle*, which is sometimes useful in dyeing, according to the nature of the shade that it imparts. The lichen strikes a brownish colour with iron, and furnishes to the Icelanders a fawn colour, with which they dye their garments. But, in Europe, our means of dyeing are so superior, that it would be wholly absurd to occupy ourselves with the lichen, under this point of view.

The infusions, of which we have spoken, are sufficient to deprive most vegetables of a portion of their nutritious principles, particularly if they be either of a gummy or saccharine nature; but the lichen does not suffer a similar loss, because its nutritious and soluble parts are very different from sugar, gum, and even from farinaceous matter.

When the lichen is boiled in water for a quarter of an hour, it becomes as tender as can be desired, and is deprived of the soluble matter which it contains.

One pound of dried lichen, after being boiled and expressed, so as to be fit for the table, yields three pounds; and I know not whether wheat, potatoes, or any other vegetable, be superior to the lichen in this respect.

Boiled lichen presents a peculiarity, which distinguishes it from all our other esculent vegetables; for whatever pressure may be employed in freeing it from the water, it immediately recovers its former bulk, in the same manner as a compressed spunge. Neither boiled spinage, nor succory, possess this quality; which most probably proceeds from the lichen not having, like other plants, that ligneous, or filamentous texture, which renders longer or shorter boiling necessary, in order to soften them. It possesses, on on the contrary, the membranous elasticity of the algæ, morels, and of some mushrooms. This quality causes the lichen very readily to yield to the least effort of the teeth; it even recalls the agreeable impression of the most tender animal cartilages.

The lichen, though resembling marine plants, has nothing, however, analogous to animal substances in its nature; whilst the algæ, on the contrary, constitute a tribe of vegetables, highly animalised in their principles.

Prepared lichen derives, besides, from its elasticity an advantage, the value of which can only be properly appreciated by those who have undertaken long voyages. It exhibits so nearly the aspect and bulk of a recent plant, that no person, who has seen it only for the first time, would venture to pronounce whether it were a raw or preserved sallad.

The boiled and dried lichen, when sprinkled with boiling water, resumes its elasticity, and is rendered immediately edible. Fresh or salt water, answers equally well for this purpose. Persons, who have eaten of it at my table, and who knew, by experience, how sensibly the privation of fresh plants is felt in long voyages, have remarked, that a stock of prepared lichen would furnish to ships-crews a fresh sallad, equally agreeable as salutary. If seafaring people are exposed to attacks of scurvy from a want of recent vegetables, we may affirm, without exaggeration, that the lichen frequently served out to ships-crews, would contribute to preserve them from this malady.

Are there any of the dried or pickled vegetables, usually stored on shipboard, which, like the lichen, possesses the advantage of supplying, without any consumption of fresh water, a sallad equally fresh as that taken from the kitchen-garden? Botanists have remarked, that lichen is not liable to suffer from the depredations of insects. Boiled and dried lichen must participate in this advantage; but if hot water restores the prepared lichen to the state of a fresh sallad, cold water also produces the same effect, but more slowly. The saving of fuel, as well as that of fresh water, which is so important at sea, renders this plant, as a sea-store, extremely valuable.

Prepared lichen, whether cold or hot, placed beneath roasted meat, or in any other way, affords a dish, of which all those, who have eaten of it on my recommendation, agree, without exception, in extolling it as extremely agreeable, and of easy digestion. I can even affirm, that a sallad of lichen has been so highly relished by all my

 friends friends, that since the period of its first introduction by me, it has been more employed as a food than a medicine.

When the lichen is boiled only for a few moments, it is equally tender as if this process had been longer continued, only it retains in that case a slight degree of bitterness. This bitterness, which resembles succory, as I have already mentioned, is not disagreeable; and if it be admitted, that it possesses a laxative quality, as Scopoli affirms, it must prove extremely salutary to many constitutions, since, according to Hoffman, the lichen *amaritie roborat, mucilagine nutrit.*

According to the opinion of several Americans, the boiled lichen has a considerable analogy to a species of fucus, which in Lima is termed *luche*, and which is very generally employed along the coast of Chili and Peru.

The boiled lichen is of an unequal dingy green hue, which is at first rather apt to convey disgust to the mind, in the same manner as the colour of chocolate is disagreeable to those who are not accustomed to its appearance; but the lichen is seldom eaten above twice, till this impression wears off, as well as that produced by its smell, which somewhat resembles the odour of marine plants.

It has been already mentioned, that a pound of dried lichen produces three pounds, after being subjected to the process of boiling. If these three pounds, after having undergone this preparation, be again dried, they become reduced to two thirds of a pound. The lichen then loses in water one third of its weight, and the water, which is absorbed and replaces this loss, amounts to two pounds and one third. Such a considerable absorption authorises us to suppose, that the pulpy portion of the lichen, though insoluble, bears a great analogy to mucilages; and perhaps, if we are to judge from the delicacy of its texture, the uniformity of its organization, as well as its membranous character, we might even suppose, that this portion is in reality only hardened mucilage.

It may perhaps be asked, if a substance, which imbibes during boiling two and one third of its weight of water, can be regarded as a solid, or nourishing food? I can only reply to this objection, as I have already done in my essay on the means of meliorating the food of soldiers.

One dozen of whites of eggs, contain in fact only one ounce of dried albumen, yet can it be supposed, that a man, who has eaten these eggs in an omelet, has not had a sufficiently substantial dinner?

Is a pound of potatoes less nourishing, from containing a largea large portion of water? The solid part of our food is not perhaps that which alone tends to repair the losses, or supply the wants of the animal system; water doubtless concurs, with the substances it decomposes, to produce this effect, by supplying two radicals, which become assimilated to the other nutritive principles.

From the above observations it is evident, that the lichen presents to us a healthy and nutritious food, and one which requires little trouble in the preparation. It were then much to be wished, that governments could be induced to diffuse a knowledge of it throughout their different countries, that the zeal of patriotic societies, and the philanthropy of individuals would contribute, by suitable encouragement, and a choice of appropriate situations, to multiply this useful vegetable; which might be advantageously cultivated in forests, on heaths, and uncultivated lands, and even perhaps upon barren rocks.

By the application of the lichen to domestic purposes, we have only discovered a part of its value. Let us now attend to the result of its analysis.

If among the leguminous vegetables, brought to our tables, there are some whose soluble parts are sufficiently agreeable to enter into the composition of soups, sauces, or be mixed with the juice of animal meats, &c. are there not many others, whose disagreeable flavour, or pungent qualities, cause them to be banished from our kitchens? What use, for example, can we make of the juice of asparagus, artichokes, potatoes, red beet, succory, and so many others? Unquestionably none, but this is not the case with the lichen: it is a plant, which nature has placed in the class of those which may be rendered doubly useful. The water in which it is boiled, is in fact, richly impregnated with nutritious substances; but as this last, though partaking of the nature of gum, forms a peculiar species, which never before resulted from the analysis of any vegetable, it is therefore necessary to treat of it more at large, in order to convey a just idea of its nature and qualities.

It has been already mentioned, that the lichen loses one third of its weight in boiling; or to speak more accurately, we may observe: first, that one hundred parts of lichen grossly pounded, yields by a cold infusion, three parts of the bitter extractive principle. Second, that one hundred parts of lichen treated with boiling water, leaves only sixty-four solid parts. The quintal of dried lichen therefore contains:

Pulp

| | | |
|---|---|---|
| Pulp | - - - | 64 |
| Bitter principle | - | 3 |
| Unknown portion | - | 33 |
| | Total | 100 |

The thirty-three parts then remain to be examined. This principle is essentially nourishing, as will hereafter be rendered evident; but is insoluble in cold water. It is then sufficient to make an infusion of the bruised moss, in order to separate the bitter extractive principle from the two others, which consist of the pulpy portion, and in the thirty-three parts of mucilaginous matter, of which we are about to speak. We have seen, that the Icelanders, in order to render the lichen palatable, are obliged to subject it to an operation, similar to that which the Indians employ in the preparation of the cassava from the roots of the manioc, and which is also practised in the kingdom of Valentia, in Spain, with the view of separating the bitter principle from the farinaceous substance of lupines. Necessity has every where taught man the art of separating a wholesome aliment from the acrid and even deleterious juices, with which it is frequently combined in vegetables. Travellers at every step, witness proofs of this industry among the most ignorant and uncivilized people.

Let us now compare the different methods employed by the Icelanders, and the inhabitants of Lapland, in the preparation of the lichen. The latter content themselves with boiling the plant, and afterwards mixing it with a certain portion of milk; but they lose, by this mode, thirty-three parts of a nutritious matter, as they throw away the water in which the plant has been boiled. The Icelanders, on the contrary, first infuse the bruised moss in cold water, by which means it is freed from the three parts of bitter extractive matter, and they thus preserve the thirty-three parts of vegetable jelly, which remain united with the pulpy substance. The Laplanders act then, if we may believe the relation of travellers, like a man who, after having boiled meat, throws away the liquor, without knowing its value.

Acquainted as we are at present with the process employed by the Icelanders, in order to convert the lichen into food, we may, without considering the qualities of the jelly, by imitating them, find in what that portion consists, which can be extracted from the washed lichen.

I boiled the farina of the washed lichen with milk, till it it appeared to be of a sufficient thickness, and afterwards seasoned it with salt and pepper, which produced an excellent dish about the consistence of rice milk, or that prepared with millet. It resembled in colour spinage diluted with milk. In this way, it forms a dish within the reach of the poor; but several persons who have eaten it, agree with me that it leaves a sense of pungency in the throat. Necessity and habit will doubtless reconcile them to this inconvenience, which is besides also found in several of the coarser foods of our own country. How many persons feel a repugnance to eat bread made of maize, on account of the austerity which is felt through its sweetness. The Indians of Brasil, accustomed to saw-dust, relish it as a most delicate food, while travellers find it only a sapless substance resembling bread which had been crumbled down for the express purpose of rendering it dry. In order to form a judgment of certain kinds of aliments, it is necessary to place ourselves, as much as possible, in the situation of those who are acquainted with no other. *Nihil contemnit esuriens.*

I next prepared another dish in the same manner, but which I seasoned with the yolk of an egg and sugar, which proved extremely palatable, and would doubtless be preferred by those in easy circumstances, but which is not more nutritive than the former. Let us now examine the jelly of the lichen.

The liquor, in which this plant has been boiled, is of a clear yellow colour, and of a somewhat bitterish taste. In cooling, it readily assumes the appearance of animal jelly; it is even of so gelatinous a nature, that a single pound of dried lichen yields on boiling eight pounds of juice, which congeals in proportion as it becomes cold.

One pound of lichen loses in a decoction only one third of its weight; this third, which, as we shall shortly see, is only a gum, runs into a gelatinous state when diffused in seven pounds and two thirds of water, or, in other words, it forms a jelly, in which the water is to the gum in the proportion of twenty-three to one. There is assuredly, in the vegetable kingdom, no other known gum, which is capable, in a given weight, of furnishing such a great mass of jelly. The animal gums alone appear equal to it in this respect.

The jelly of the lichen is besides distinguished by characters, which appear peculiar to it. If set aside, for a single day, the water begins to separate round its edges, however cold may have been the place in which it was kept.

If

If we incline the dish in different directions, it cracks and splits with a facility which we never observe either in animal or vegetable jellies; and in the fissures we observe that the water augments, and tends to separate from the jelly, or that the latter abandons it, probably in consequence of the little affinity which prevails between the water and the gummy principle of the lichen.

During the boiling of the jelly, the gummy matter forms at the surface transparent pellicles, which are renewed in proportion as they are skimmed off, so that we may collect in this way the greatest part of the gum; but these pellicles when sunk again in the liquor are not re-dissolved in it, unless it be at the boiling point; a circumstance which corroborates the opinion that this species of mucilage is in reality less soluble than any of those yet known in the vegetable kingdom.

The jelly of lichen, when sufficiently inspissated, does not become viscous either by cold or heat; in this respect it resembles neither any of the gums, nor any of the mucilages; it shews not the least adhesive quality, and hence it appears doubtful whether it may prove useful in any of the arts, except, perhaps, in printing linen; this, however, is a point which demands farther investigation. It follows also as a consequence of its want of viscidity, that this jelly, dried upon dishes, separates into transparent, angular, and brittle fragments, the shade of which is of a deep red. Animal or vegetable jellies are in this respect extremely different, because from the coherence of their parts, they dry without experiencing any separation.

If we throw into cold or warm water the desicated gum of the lichen, it is not dissolved; it softens and swells without becoming gluey or glary; but it also deposits very quickly all its extractive principle, and consequently loses its bitterness, which may suggest a means of purifying it, if hereafter it should be found to possess any property which may render it of additional value. The pellicles, as well as the gum, require boiling water to dissolve them; and upon cooling, we procure the jelly destitute of bitterness.

The infusion of galls, which exerts no action upon any of the gums hitherto known, instantaneously precipitates the jelly of lichen, and produces a white coagulum as with the animal jellies; but with this difference, that the new combination is soluble in water, and separates from it on cooling. This then is the distinctive character of the mucilage of lichen. A similar quality would seem to indicate some some relation between this mucilage and that of animals; but the following experiments invalidate this opinion. The gum of the lichen heated in a cucurbit, is destroyed without becoming softened. Like gum arabic it leaves only from 23 to 24 centiemes of carbon. Its products do not differ from those of gum or starch, consisting of water and acetous acid of the same odour; but the oil appears to be much more abundant. Potash separates from this acid a few particles of ammonia.

The nitric acid readily converts it into a very white oxalic acid. The residuum contains neither unctuous matter, nor the bitter principle. Boiled and dried lichen yields only of carbon, from 21 to 22 centiemes.

Nitric acid dissolves boiled lichen with much facility, a circumstance not common with ligneous vegetables. The products are oxalic acid and oxalate of lime, but apparently augmented by some unknown earth. The residuum contains only a very small portion of the bitter principle.

Potash converts boiled lichen into a sort of gelatinous pulp, very similar to that yielded by farinaceous matter in like circumstances. This sufficiently shews that the pulpy portion of the plant is nothing more than an indurated gum, less oxyginized, perhaps, than those which are soluble. Nature, it should seem, has rendered more than usually nutritious this vegetable, which is intended to supply the place of so many others, in those regions where the severity of the cold benumbs the powers of vegetation.

The jelly of lichen properly prepared, may be rendered very agreeable. The extractive part which it contains, is neither so considerable, nor so bitter as not to admit of being concealed. The following formula, among others, seems well calculated to answer this intention.

Take of lichen, four ounces; water, three pounds; boil it down to two; after which, add to it of flour, one drachm; sugar, four ounces. Next take of sweet almonds, No. 60; bitter almonds, No. 24; citron peel, a small quantity. After rubbing these well together, moisten the paste with a few spoonfuls of warm water, in order to dispose it to yield its milk. Mingle this with the jelly while hot; strain it through a cloth previously moistened with boiling water; and, lastly, pour it into small tart pans.

The intention of adding the flour, is to give to the jelly greater consistence, and to diminish its tendency to crack and separate. The odour and taste of the bitter almonds, effectually cover and conceal the bitterness of the lichen. In cases where it is proper to prescribe to patients the jelly of of lichen, this unquestionably is the form which appears to me to claim a preference above all others.

If the jelly of the lichen, thus seasoned, cannot be considered as a very powerful medicine, it nevertheless partakes in common with all animal and vegetable jellies, of a highly restorative quality. Some may, perhaps, express surprise that this gum should require condiments to render it digestible. It may be observed, however, that this inconvenience is not peculiar to it, but extends to all other gelatinous products, whether animal or vegetable, which constitute the principle basis of our aliments. Starch is the basis of bread. Strong glue constitutes that of soup. What stomach could support the jelly of starch, of bones, of hartshorn, &c. if these insipid mucilages were not seasoned by savoury and aromatic ingredients. The use of condiments is founded much more upon the necessity of stimulating the stomach, and promoting digestion, than upon that of pleasing the palate.

The gum of lichen dried and diluted in much water, may also supply us from what we have seen, with jellies of a more simple kind. The lichen itself, bruised and deprived of its bitter principle, may likewise be employed for this purpose.

In dilating so much as I have done on this topic, I am not ambitious of introducing a new alimentary substance to the detriment of others. While there are hartshorn shavings, the lichen will probably not be resorted to as the basis of white jellies; but I have thought it incumbent on me to show the useful purpose to which this plant might be applied in cases of general scarcity.

In short, the gelatinous product of lichen may henceforth be considered as a new species in the family of gums. The property which it possesses of only being soluble in boiling water, seems to indicate some analogy with starch; while, on the other hand, if we recall to mind that it possesses no viscosity, that it cannot be converted into glue, it must be evident that there is much difference between them. It would nevertheless be worth while to subject the lichen to fermentation, in order to ascertain whether starch might not be separated from it; for in this case, its utility would be greatly extended.

But in a family which contains so many species as that of the lichen, *nimis vastum genus*, we may reasonably suppose, that nature has not confined herself to accumulate so much nutritive matter in one of these same species exclusively, and that there doubtless must be others which possess sess sess this advantage with the Islandic lichen. Hence it would be proper to extend our researches to these, as botanists may meet with them. Georgi, a learned Russian, has already entered on this investigation, as appears by what he has written in the Transactions of the Academy of Petersburgh for the year 1779. Georgi, in fact, discovered that the lichens termed *physoïdes, hirtus, farinaceus et pulmonarius,* yield a very mucilaginous fluid, and that the boiled plants may be eaten. *Et ipsi lichenes addito sale, edules fiebant;* from whence he concludes, that the poor would find in them, *urgente annonæ difficultate,* an inexhaustible resource.

These advantages are fully confirmed by the plump state of the rein-deer, which, as is well known, derives its subsistence from the lichen, which it digs from under the snow, and the want of which no other aliment can supply to it in this situation. On boiling one of the varietes of the *rangiferus* which is contained in the King's park, near Madrid, it yielded only a very small portion of jelly, which, however, did not appear to be different from that of the lichen islandicus, but the plant never became soft. Botanists ought to endeavour to ascertain what means are necessary to extend the propagation of the lichen islandicus, by transplanting it into situations most analogous to those in which it is found to thrive best. It will not be difficult to determine the local circumstances which may contribute to the multiplication of a plant so extremely interesting to mankind.

I am, &c.

H——.

*December* 7, 1807.

